# Investigating the effects of mycoprotein and guar gum on postprandial glucose in type 2 diabetes: a double-blind randomised controlled trial

**DOI:** 10.1038/s41387-025-00375-w

**Published:** 2025-05-23

**Authors:** Anna Cherta-Murillo, Kexin Zhou, Martina Tashkova, James Frampton, Ana Cláudia Cepas de Oliveira, Claire Ho, Georgia Franco-Becker, Edward S. Chambers, Anne Dornhorst, Gary S. Frost

**Affiliations:** 1 Section for Nutrition Research, Department of Metabolism, Digestion and Reproduction, Faculty of Medicine, Imperial College London, Hammersmith Hospital, London, W12 0NN UK; 2 Imperial College Healthcare NHS Trust, Hammersmith Hospital, London, W12 0NN UK

**Keywords:** Type 2 diabetes, Randomized controlled trials

## Abstract

**Background:**

Type 2 diabetes (T2D) is highly prevalent, particularly among south Asian populations, and diet is the first-line strategy to manage postprandial glucose (PG) response. Mycoprotein and guar gum reduce PG in normo-glycaemic people. This study investigates the independent and interactive effects of mycoprotein and guar gum on PG, insulin and appetite responses in white Europeans and south Asians with T2D.

**Methods:**

In this double-blind, crossover, acute, randomised controlled trial, 18 subjects with T2D (10 white European, 8 south Asian) completed six separate visits consuming soy, chicken, and mycoprotein with and without guar gum. Incremental area under the curve (iAUC_0-180 min_) for PG, insulin, and appetite scores, and total AUC_0-180 min_ glucagon-like peptide-1 (GLP-1), peptide tyrosine-tyrosine (PYY), as well as *ad libitum energy intake* and 48h-post-visit energy intake were measured and analysed by linear mixed models with protein, guar gum and ethnicity as fixed effects.

**Results:**

We found independent effects of mycoprotein, guar gum and ethnicity on PG iAUC_0-180 min_ (mmol/L·min), where mycoprotein reduced PG vs. chicken (–129.84 [95% CI –203.16, –56.51]; *p* = 0.002), guar gum reduced PG vs*.* no guar gum (–197.35 [95% CI –254.30, –140.40; p < 0.001], and south Asian had increased PG vs. white Europeans (195.75 [95% CI 66.14, 325.35]; *p* = 0.005). An interaction between guar gum and ethnicity (*p* < 0.015) was found for insulin iAUC_0-180 min_ (µUI/mL·min), with guar gum lowering insulin responses in south Asian participants (–1909.69 [95% CI –2834.83, –984.511]; *p* < 0.001). No independent or interactive effects were observed for appetite-related outcomes.

**Conclusion:**

Mycoprotein and guar gum promote significant independent effects in lowering PG in both white European and south Asians with T2D.

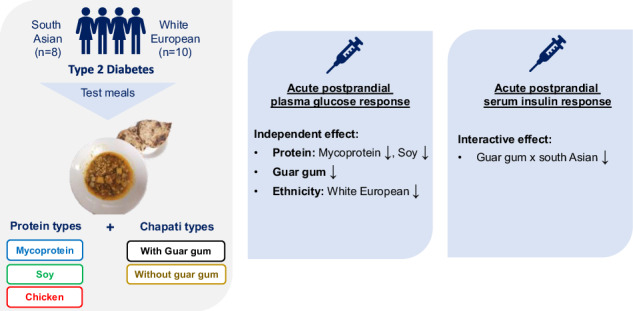

## Introduction

Type 2 diabetes (T2D) affects approximately 420 million adults globally and is predicted to increase to ~700 million by 2045 [1]. Prevalence rates are high among South Asian adults compared to white Europeans (25% vs. 5%, respectively) [2]. Over time, high postprandial glucose (PG) increases the risk of microvascular and macrovascular complications, including atherosclerosis-related diseases (e.g. coronary heart disease), kidney failure, retinopathy, and limb amputations [3]. The prevalence of these complications is higher in South Asian populations as compared to other regions [4].

Dietary management remains the first-line treatment strategy for T2D [5]. Food with high glycaemic load or high in available carbohydrates are the main contributors to increased PG response [6]. Interestingly, a limited number of studies have shown differences in metabolic response between ethnicities when fed the same intervention. A study administrating a 5-day dietary challenge mimicking western-diets (high fat, high-calorie diet) showed that healthy South Asians exhibited increased fasting glucose, insulin and insulin resistance compared to white European counterparts [7]. Furthermore, another study in healthy adults showed that the PG response was significantly higher in South Asians (e.g., by +62% for malted wheat cereal, high in fibre) compared to matched-white Europeans [8]. However, to our knowledge, data on the PG response to an acute dietary challenge in South Asians and white Europeans with T2D is not available. Thus, nutritional solutions or ingredients which adapt to one’s cultural preferences, that lower PG response in people with poor metabolic control, are of great need. In this context, mycoprotein is a protein-rich ingredient derived from the continuous fermentation of the fungus *Fusarium venenatum* [9] (nutritional profile shown in Supplementary Table 1) which is currently available in 16 countries as meat-alike products (available from Quorn^TM^). Mycoprotein has been shown to improve glycaemic control, lipid profiles and to lower appetite and energy intake, in both acute and in mid-term (7-day) interventions [10–13] in subjects with normal glycaemic control. These effects have been attributed to its intrinsic fibre and protein content, as well as its unique food structure (i.e. hyphae), including its fungal cell wall, which makes alpha-amylase diffuse into it, entrapping it, being less available to perform carbohydrate enzymatic hydrolysis, as observed in vitro [14]. Evidence shows that high dietary fibre and protein intake play a role in regulating glycaemia and appetite responses [15–17], achieved through elevated incretin secretion [18], insulin secretagogue effects [19], and the stimulation of anorexigenic gut hormones such as glucagon-like peptide-1 (GLP-1) and peptide tyrosine tyrosine (PYY) [20]. Nevertheless, to date, no study has sought to examine the effect of mycoprotein on postprandial responses in people with T2D.

Other ingredients with relevant efficacy at decreasing PG response and appetite include guar gum, which is a soluble fibre derived from processed guar bean (*Cyamopsis tetragonoloba)*. The effects of guar gum on the glycaemia and appetite responses have been tested in people with T2D [21–23], and have been mechanistically linked to delayed gastric emptying, owing to the viscous qualities of guar gum [24]. We therefore hypothesised that guar gum and mycoprotein would lower PG response, and may act synergistically when combined, in subjects with T2D. The aim of our work was to determine the independent, and interactive effects of mycoprotein, guar gum and ethnicity in lowering both PG and insulin responses, as well as appetite responses in people with T2D.

## Materials and methods

### Subjects

A total of 25 non-insulin treated male and female subjects with T2D (12 white Europeans and 13 South Asian) volunteers were recruited from May 2019 until February 2021 (with a pause from March 2020 to September 2020 due to COVID-19 pandemic). Recruitment was performed via advertisements in the press, community posters (including local community NHS organizations and GP practices) and social media. Participants were screened against the eligibility criteria. Inclusion criteria consisted of being diagnosed with T2D, being of South Asian origin (Afghanistan, Bangladesh, Bhutan, Maldives, Nepal, India, Pakistan, and Sri Lanka) or white European ethnicity (ethnicity was self-reported as origin of the 4 grandparents), aged 18-70 years old, and having 5.5 ≤ HbA1c ≤ 9.0%.

Exclusion criteria included: parents of different ethnicity (self-proclaimed), insulin therapy, GLP-1 agonists or orlistat, gastrointestinal disease effecting bowel function or nutritional intake, significant heart, hepatic or renal disease (requiring dialysis), cancer, pancreatitis, history of alcohol and/or drug abuse, or current smokers. Participants were also excluded if they were shift workers, or people taking acarbose, meglitinides, insulin, or systemic glucocorticoids within 2 weeks prior to study entry, or antibiotics within 3 weeks, weight change of ≥5% in the preceding 3 months.

### Study design

**Fig. 1:**
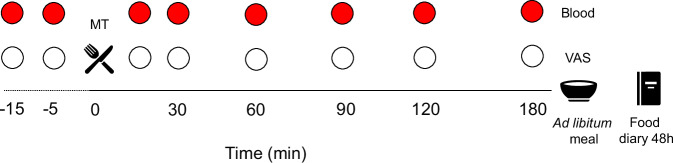
Timeline of a study visit. Circles represent a timepoint of measure collection. TM test meal, VAS visual analogue scale for subjective appetite feelings; min, minutes.

In this double-blind, crossover, acute, randomised controlled study, 18 participants completed the study through 6 separate study visit days. The interventions (test meals) were blinded for both participants and the study researcher thanks to their similar appearance, palatability, and weight. Moreover, the meal had coded labelling for blinding purposes. Those involved in sample and statistical analysis were also blinded. The study visit order were randomised using www.sealedenvelope.com. The same researcher generated the random allocation sequence, enroled participants, and assigned participants to interventions. Visits were separated by a wash-out period of 3–7 days. Participants were asked not to change their physical activity routine or dietary habits throughout the study, but were asked to refrain from strenuous physical exercise for 72 h prior to the study visits. Participants attended the NIHR Imperial Clinical Research Facility at Hammersmith Hospital, London, UK, following an overnight fast. The last dose of any oral medication was to be taken 8 h the day before each study visit and not during the test meal of the study visit. 24 h prior to the study visit participants were asked to consume a standard evening meal of their choice, which was kept constant on the following visits, and monitored by asking the participants to provide the package label at each visit. Participants were asked to abstain from alcohol for 24 h prior to each study visit. Figure 1 illustrates the study visit timeline of study procedures.

Upon arrival, body weight and composition were measured with a bioelectrical impedance scale (Tanita^®^, BC-418 analyser, Tanita Corporation, Japan) following blader emptying. Next, an intravenous cannula for serial blood sampling was placed into the antecubital fossa, which was kept patent for 195 min to allow blood withdrawal at different timepoints without further discomfort. This allowed for 2 fasting (–15 and –5 min relative to start of meal intake) blood samples. Then, a test meal (details in the Test Meal section) was served and asked to be consumed within 15 min, with both researcher and participants blinded to the type of meal. Next, participants rated its palatability with an analogue scale ranging from 1 (not good at all) to 10 (extremely good). Seven postprandial 8 ml blood samples were drawn at 15, 30, 45, 60, 90, 120, and 180 min relative to start of meal intake. Before every blood sample, participants completed a 100 mm visual analogue scale (VAS) to assess subjective appetite feelings (Supplementary Table 2). After the last blood sample, the cannula was removed, and participants were asked to consume an *ad libitum* meal consisting of excess homogenous pasta with tomato sauce and olive oil (Sainsbury’s, UK) to assess *ad libitum* energy intake. Participants were instructed to eat until they felt “comfortably full,” and to avoid any distractions (e.g., electronic devices such as mobile phones) while eating. Participants were told at screening that this test’s purpose was to assess their appetite and energy intake and that pasta would be measured before and after consumption.

Finally, participants were asked to keep a food diary of all dietary intake for the next 48 h to assess energy intake post-visit.

### Test meals

**Fig. 2:**
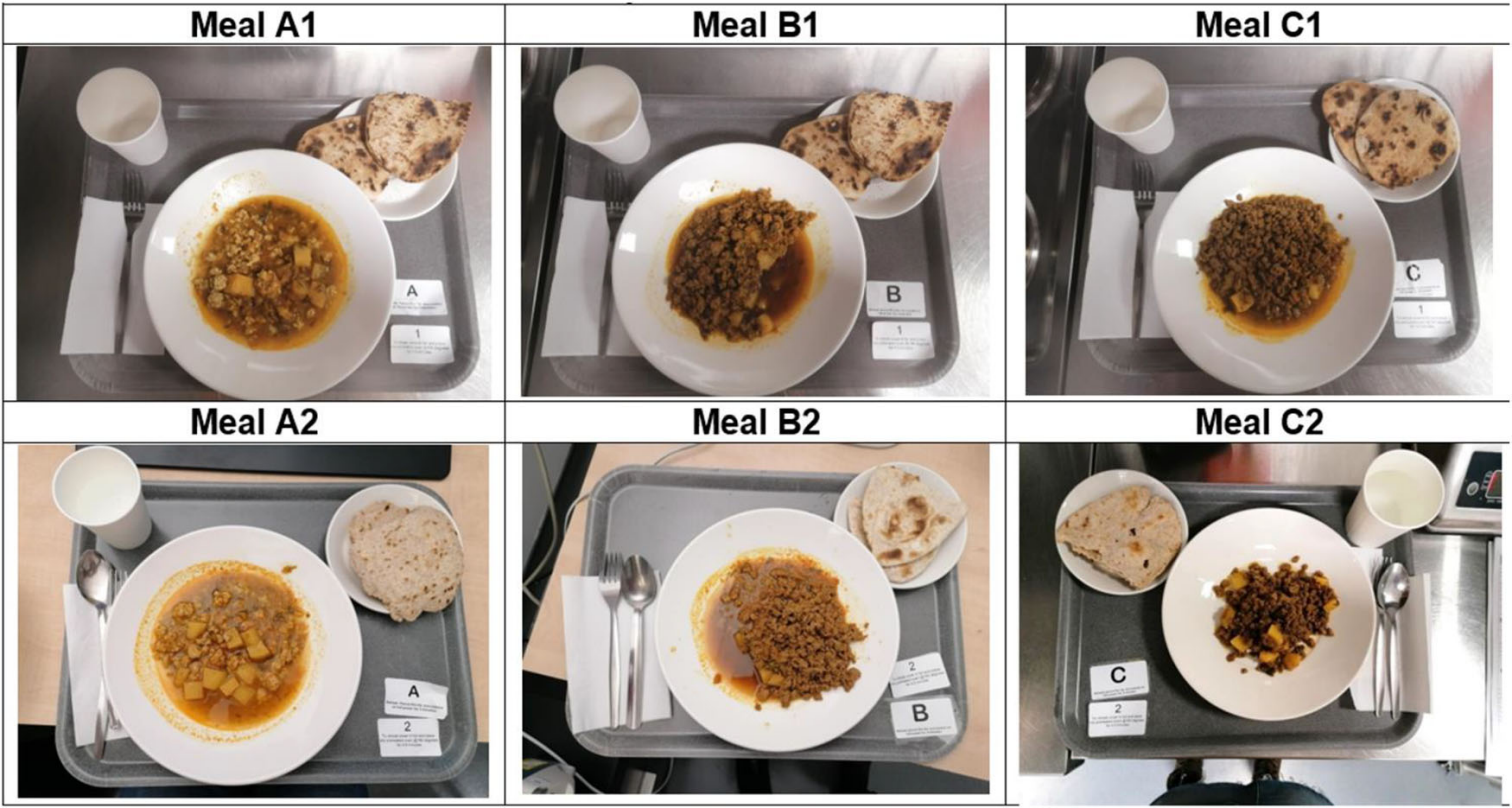
Pictures of the six meals served on six different occasions during the study. **A** Chicken, (**B**), mycoprotein, (**C**), soy, 1, plain chapati, 2, guar gum-enriched chapati.

Participants were given the following six energy and macronutrient-matched test meals: (a) soy mince with guar gum-enriched chapati, (b) chicken mince with guar gum-enriched chapati, (c) mycoprotein mince with guar gum-enriched chapati, (d) soy mince with plain chapati, (e) chicken mince with plain chapati, (f) mycoprotein mince with plain chapati. Guar gum-chapatti was prepared with 100 g of flour enriched with 5 g of guar gum (5%), previously demonstrated improvements in glycaemic control [25]. The chapati without guar gum had the same amount of flour (100 g) and was within 5% similitude of carbohydrate content as the guar gum-enriched chapati Chapatis, are a staple food in South Asian population, and its enrichment with guar gum was tested previously [22]. All tests meals were specifically prepared for this research study by NPD Ltd (UK). The six meals had a similar appearance, palatability, and weight (Fig. 2). They were served after heating in the microwave according to the manufacturer’s instructions. Table 1 describes the nutrient profile of the test meals. More details on the ready meals’ manufacturing, preparation and blinding, as well as the nutritional profile of the plain chapatis can be found in Supplementary Table 3.

**Table 1:** Nutritional composition of the complete test meals with soy, chicken and mycoprotein- based mince meals (including guar gum-enriched chapati).

Per serving	Soy with GG-enriched chapati (400 g)	Chicken with GG-enriched chapati (400 g)	Mycoprotein with GG-enriched chapati (350 g)
Energy (kcal)	473	461	461
Carbohydrates (g)	57	58	55
Fat (g)	7	10	9
Protein (g)	36	31	30
Fibre (g)	19	11	19

GG-enriched chapati was enriched with 0.5 g of GG per 10 g of flour (100 g of flour in total) as similar to Ellis and colleagues [35].

*G* gram, *GG* guar gum.

### Measurement of plasma glucose, insulin, gut hormones, appetite responses and energy intake

At screening, blood tests were performed to assess full blood count, fasting glucose, HbA1c, liver function, and lipids. All were analysed as per standard hospital pathology laboratory procedures. Collected blood samples during the study visits were analysed for plasma glucose, serum insulin, plasma GLP-1 and PYY. All study visits’ blood samples underwent centrifugation at 2500 × *g* for 10 min. Serum (after clotting) and plasma were separated after centrifugation. Aliquoted samples were immediately stored at -80^o^C for later analysis. Biochemical analysis of plasma samples for glucose was performed using a commercially available enzymatic colorimetric assay kit (Glucose GOD/PAP GL 364, Randox Laboratories Ltd, UK). Biochemical analysis of the serum samples for insulin concentrations was performed using a quantitative commercially available assay kit named Human Insulin Specific RIA Kit (HI-14K, Sigma Aldrich, Merck, Germany). All assays were performed according to the manufacturer’s protocol. Total GLP-1 and total PYY were measured using established in-house radioimmunoassays [26, 27]. The inter-assay and fasting coefficient of variation and the mean (SD) fasting values for the blood metabolites were reported in the Supplementary Tables 4, 5. Subjective appetite feelings were measured with the use of 100 mm VAS [28]. For *ad libitum* energy intake, the amount of food (grams) eaten was quantified using a scale and energy content was calculated. For post-visit energy intake, participants were asked to fill a 48 h food diary record for energy intake assessment. Average daily energy and macronutrient intake was calculated using Dietplan 6 (Foresfield Software Ltd, West Sussex, UK).

### Power calculations

A power calculation was not possible as no previous literature existed which included people with T2D being fed acutely with mycoprotein or similar food of nutritional equivalence. While literature on the effect of mycoprotein in healthy subjects (not with prediabetes nor with T2D) was available, this data was deemed inappropriate to be used to power this study as the PG in subjects with T2D is greater than of subjects with normal glucose tolerance [29]. Hence, we chose a sample size of 12 per group, as recommended for unfounded studies [30], and justified based on feasibility, precision about the mean and variance, as well as regulatory considerations in food trials. Because we also we wanted to explore the independent and synergistic effect of ethnicity as one of the factors, we aimed to recruit 12 subjects of each ethnicity (i.e., 12 South Asian and 12 white Europeans), totalling 24.

### Statistical analysis

The primary outcome was PG response via incremental area under the curve (iAUC) 0–180 min in all subjects, exploring the independent and interactive (or synergistic) effects of protein, guar gum or ethnicity. The secondary outcomes were postprandial insulin iAUC_0-180_, GLP-1 and PYY tAUC_0-180_, *ad libitum* energy intake, 48 h post-visit energy intake, and subjective appetite iAUC_0-180_.

Linear mixed models were used to evaluate the independent and interactive effects of protein (soy, chicken, and mycoprotein) guar gum (with- and without) and ethnicity (white European and South Asian) on all outcomes. Fixed effects included in the model were protein, guar gum, and ethnicity. Random effects included in the model were participant. Where a significant main or interaction effect was detected, post-hoc multiple comparisons were performed with the Tukey's test. Independent effects were defined as a significant main effect of protein and/or guar gum and/or ethnicity in the absence of a significant interaction effect. Interactive effects were defined as a significant interaction effect between any of the 3 factors protein, and/or guar gum and/or ethnicity [31]. A statistical significance was determined at *P* ≤ 0.05. Statistical analyses were performed using JASP (Version 0.18.3) (Amsterdam, The Netherlands). Statistical analyses were carried out using a blinded outcome assessor, meaning the analysis was unblinded only after the analysis was completed.

Results are presented as means ± standard deviation (SD) in Figures, and when in narrative as mean and 95% confidence interval (CI) for glucose, insulin, subjective appetite, and energy intake. The average of two separate baseline measurements (–15 and –5 min relative to meal intake) for plasma glucose, insulin and VAS data were used for the 0 min measurement. For plasma glucose and insulin data, the incremental area under the curve (iAUC) 0–180 min was calculated using the trapezoidal rule. Gut hormone data was analyzed using the total AUC (tAUC_0-180_) to inform on the total hormonal output during the test meal. For the subjective appetite data, a composite appetite score (CAS) was calculated by averaging hunger, desire-to-eat, prospective food intake and 100-fullness [32]. The iAUC_0-180_ of CAS and sickness VAS was analysed. Graphs were prepared using GraphPad Prism v9.0 (GraphPad Software, San Diego, CA, USA).

This study was conducted according to Declaration of Helsinki’s guidelines, and all procedures involving human subjects were approved by Bromley Research Ethics Committee (19/LO/0476). Written informed consent was obtained from all subjects. The trial was registered on www.clinicaltrials.gov (NCT03949582).

## Results

### Subject’s baseline characteristics

The CONSORT diagram of subjects’ flow for crossover studies is described in Supplementary Fig. 1. Eighteen participants completed the study (*n* = 10, white Europeans, and *n* = 8, South Asian).

The demographic baseline characteristics of the participants are outlined in Table 2. Supplementary Table 6 represents the baseline energy and food intake of participants.

**Table 2:** Demographic characteristics of 18 participants (11 white European and 10 South Asian).

	Total (*N* = 18)		White European (*n* = 10)		South Asian (*n* = 8)		*P* value
	Mean	SD	Mean	SD	Mean	SD	
**Age**	61	7.65	62	7.63	59	7.65	0.45
**Sex (male/female)**	–		11 (10/1)		10 (9/1)		–
**Body weight (kg)**	78.26	16.41	88.55	15.06	66.93	8.60	<0.01
**BMI**	26.00	3.48	27.70	3.28	24.12	2.75	0.01
**Ethnic-specific BMI categories**							
**Healthy (%)**	–	–	18		30		–
**Overweight (%)**	–	–	54		50		–
**Obese (%)**	–	–	27		20		–
**Body fat (kg)**	19.36	6.87	22.85	6.83	14.45	2.66	<0.01
**Body fat (%)**	24	4.54	25	4.94	22	2.72	0.06
**Fat-free mass (kg)**	58.14	10.95	62.45	9.82	52.17	8.89	0.02
**Fat-free mass (%)**	71	7.88	71	4.74	70	9.96	0.91
**Visceral fat score**	11.28	2.43	12	2.59	10.22	2.25	0.21
**Serum HDL cholesterol (mmol/L)**	1.22	0.27	1.29	0.27	1.13	0.22	0.19
**Serum LDL cholesterol (mmol/L)**	2.23	0.73	2.26	0.83	2.18	0.70	0.81
**Serum TAGs (mmol/L)**	1.04	0.37	1.37	0.40	0.85	0.35	0.01
**Serum ALTs (IU/L)**	27.75	12.88	30	15.42	25	10.85	0.40
**Fasting plasma glucose (mmol/L)**	7.39	1.42	8.12	0.23	6.48	0.25	<0.01
**HbA1c (mmol/mol)**	52.76	7.10	51.82	5.44	53.80	8.76	0.53
**Years since T2D diagnosis**	6.99	5.18	6.32	4.31	7.73	6.17	0.54
**T2D management**							
**Only lifestyle (%)**	10		18		0		-
**Metformin (%)**	86		73		100		-
**Sulfonylurea (%)**	29		27		30		-
**DPP4i (%)**	19		27		10		-
**SGLT-2i (%)**	10		18		0		-

Visceral fat score: fat in the internal abdominal cavity, surrounding vital organs in the trunk. Rating from 1-12 means healthy levels whereas levels 13-59 indicate an excess level of visceral fat. Bioelectrical impedance analysis correlates with MRI-defined VAT (r = 0.74, *P* < 0.001) [43]. *T* test performed between South Asians and white Europeans. *P* value ≤ 0.05 is considered significant.

*BMI* body mass index, *DPP4i* dypeptidyl 4 inhibitor, *HbA1c* glycated A1c haemoglobin, *IU* international units, *SD* standard deviation, *SGLT-2i* sodium glucose transporter 2 inhibitor, *T2D* type 2 diabetes.

### Palatability of the test meals

The palatability score (from 1, not at all palatable, to 10, extremely palatable) ranked by participants immediately after consuming the test meals were (mean ± SD): soy (6.69 ± 0.45), chicken (7.81 ± 0.32), and mycoprotein (5.68 ± 0.99) with plain-chapati and soy (7.13 ± 0.57), chicken (7.81 ± 0.35) and mycoprotein (5.68 ± 0.39) with guar gum enriched-chapati. No independent effect of protein (*p* = 0.54), guar gum (*p* = 0.89), ethnicity (*p* = 0.70), nor any interaction, was detected.

### Postprandial plasma glucose response

**Fig. 3:**
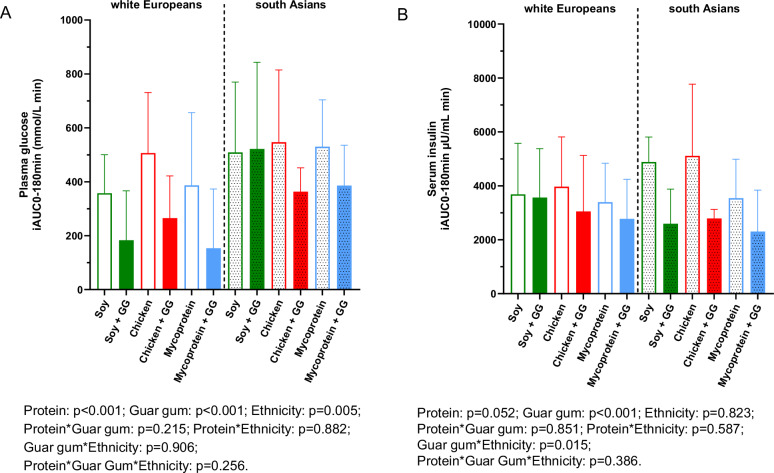
Plasma glucose and serum insulin iAUC0-180min following consumption of soy, chicken, and mycoprotein with or without guar gum for all participants (both South Asians and white Europeans) (n = 18). Plasma glucose (**A**) and serum insulin (**B**) iAUC_0-180_ following the consumption of soy, chicken and mycoprotein with or without guar gum for all participants (both South Asians and white Europeans) (*n* = 18). Data represents mean ± SD. The colour scheme represents the type of proteins, these being soy (green), chicken (red), and mycoprotein (blue). The filling of the boxes represent the type of chapatis, these being without guar gum (empty boxes) and with guar gum (filled boxes). Dotted boxes represent South Asians, while non dotted represent white Europeans. The *p* values from linear mixed models for independent effects of protein, guar gum and ethnicity and for the interaction between them are presented. GG guar gum, iAUC incremental area under the curve, n sample size, SD standard deviation.

We performed linear mixed model analyses to identify the independent and interactive effects of protein type (soy, chicken and mycoprotein), guar gum (with- and without), and ethnicity (South Asian or white European) on PG responses (Fig. 3). Temporal PG responses from 0 to 180 min are presented in Supplementary Fig. 2. We found independent effects of protein (*p* < 0.001), guar gum (*p* < 0.001) and ethnicity (*p* < 0.005), with no significant interaction between factors. Looking at the independent effect of protein type, PG iAUC0-180 min were significantly lower by –27% for mycoprotein compared to chicken (mean difference (MD) –129.84 mmol/L·min; [95% CI –203.16, –56.51]; *p* < 0.002), and by –31% for soy compared to chicken (MD: –151.62 mmol/L·min; [95% CI –225.23, –78.01]; *p* < 0.001). PG iAUC0-180 min was not significantly different between soy protein and mycoprotein (MD: –21.79 mmol/L·min, [95% CI –87.79, 44.22]; *p* = 0.885).

The independent effect of including guar gum, regardless of protein or ethnicity, significantly decreased PG iAUC0-180 min response by –40% (MD: –197.35 mmol/L·min; [95% CI –254.30, –140.40]; *p* < 0.001).

When testing the independent effect of ethnicity, PG iAUC0-180 min response of the South Asian participants was +40% greater (MD: 195.75 mmol/L·min; [95% CI 66.14, 325.35]; *p* = 0.005) compared to the white European participants.

### Postprandial serum insulin response

For insulin iAUC_0-180_ (Fig. 3), there was no statistically significant independent effect of protein (*p* = 0.052). There was a significant interaction between guar gum and ethnicity (*p* = 0.015). A post-hoc analysis for multiple comparisons showed that guar gum only lowered postprandial insulin in the South Asian participants (–1909.69 μIU/mL·min [95% CI –2834.83, –984.511]; *p* < 0.001) and not the white European counterparts (–527.23 μIU/mL·min [95% CI –1123.11, 69.95]; *p* < 0.157). Temporal insulin responses from 0 to 180 min are represented in Supplementary Fig. 3.

### Postprandial gut hormone response

**Fig. 4:**
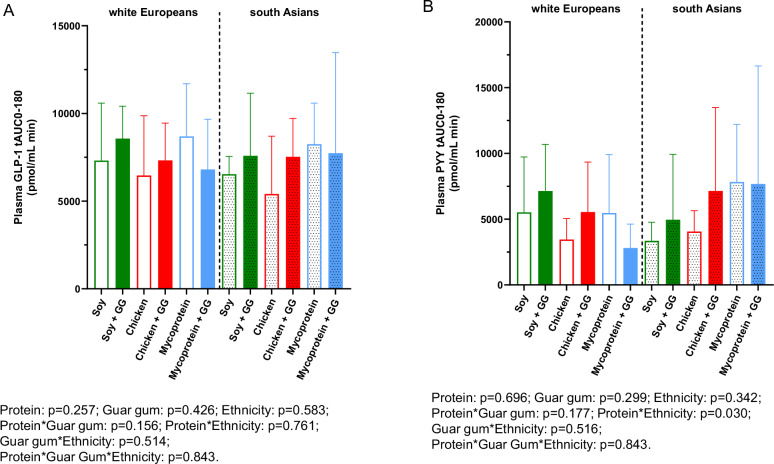
Plasma total GLP-1 and PYY total AUC_0-180min_ following consumption of soy, chicken, and mycoprotein with or without guar gum for all participants (both South Asians and white Europeans) (*n* = 18). **A** GLP-1 total AUC_0-180_ and **B** PYY total AUC_0-180_. Data represents mean ± SD. The colour scheme represents the type of proteins, these being soy (green), chicken (red), and mycoprotein (blue). The filling of the boxes represents the type of chapati, these being without guar gum (empty boxes) and with guar gum (filled boxes). Dotted boxes represent South Asians, while non dotted represent white Europeans. A linear mixed models for independent effects of protein, guar gum and ethnicity and for the interaction between them was performed and showed no significances. GG, guar gum; GLP-1, glucagon-like peptide-1; n, sample size; SD, standard deviation; tAUC, total area under the curve.

The plasma GLP-1 and plasma PYY total AUC_0-180_ for all participants are presented in Fig. 4 and its temporal gut hormone responses from 0 to 180 min are represented in Supplementary Fig. 4. For total plasma GLP-1 AUC_0-180_, there were no independent effects of protein type (*p* = 0.257), guar gum (*p* = 0.426), and ethnicity (*p* = 0.583), nor any interactive effect. For total plasma PYY AUC_0-180_, there were no independent effects of protein type (*p* = 0.696), guar gum (*p* = 0.299), and ethnicity (*p* = 0.342), however, there was a statistically significant interactive effect of protein type and ethnicity (*p* = 0.030). Multiple comparisons showed no significant differences when comparing protein type and ethnicity combinations.

### Energy intake at *ad libitum* and 48-h post-visit

**Fig. 5:**
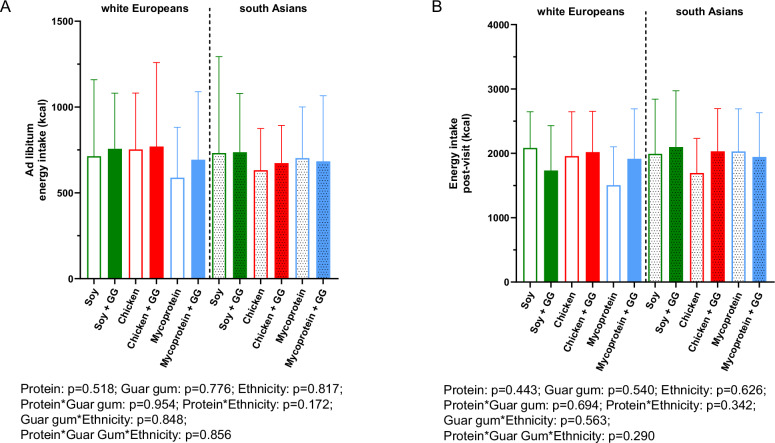
*Ad libitum* and post-visit energy intake following consumption of soy, chicken, and mycoprotein with or without guar gum for all participants (both South Asians and white Europeans) (*n* = 18). **A** *Ad libitum* energy intake and (**B**) post-visit energy intake per day following test meals. Data represents mean ± SD. The colour scheme represents the type of proteins, these being soy (green), chicken (red), and mycoprotein (blue). The filling of the boxes represents the type of chapati, these being without guar gum (empty boxes) and with guar gum (filled boxes). Dotted boxes represent South Asians, while non dotted represent white Europeans. A linear mixed models for independent effects of protein, guar gum and ethnicity and for the interaction between them was performed and showed no significances. GG, guar gum; kcal, kilocalorie; n, sample size; SD, standard deviation.

The effect of the test meals on *ad libitum* and post-visit (48 h) energy intake is presented in Fig. 5. For the *ad libitum* energy intake, no statistically significant independent effect of protein (*p* = 0.518), guar gum (*p* = 0.776), ethnicity (*p* = 0.817), nor interactive effects were found. Similarly, for the 48-h post-visit energy intake, no statistically significant independent effect for protein (*p* = 0.443), guar gum (*p* = 0.540), ethnicity (*p* = 0.626), nor interactive effects were found.

### Subjective appetite feelings

There was not a statistically significant main independent effect of protein type, guar gum, ethnicity, or interactive effect for CAS or sickness (*p* values not shown) (Supplementary Fig. 5). CAS and sickness VAS temporal responses up to 180 min are represented in Supplementary Fig. 6.

## Discussion

This study investigated the independent and interactive effects of mycoprotein and guar gum (within a mixed meal providing approximately 60 g of carbohydrate) on acute postprandial glucose, insulin, gut hormone responses, subjective appetite, and energy intake in adults with T2D of South Asian and white European ethnicities. We found that PG response was modulated by independent or additive effects of protein, guar gum and ethnicity, where mycoprotein independently reduced PG response by -27% (similarly to soy) vs. chicken. Moreover, we found that guar gum independently reduced PG response by –40%, however, there was no interactive effect between protein and guar gum. For postprandial insulin, an interactive or synergistic effect of guar gum with ethnicity was found, where guar gum only reduced insulin levels in the South Asian participants.

Evidence in the literature on the effects of mycoprotein on postprandial glucose and insulin response iAUC0-180 min is limited to only one study including healthy subjects with overweight and obesity [33], in which similar amounts of mycoprotein (132 g) to the ones tested in the present study, did not lead to a decrease in glucose iAUC0-180 min but decreased insulin iAUC0-180 min by 21% compared to protein and energy matched-chicken combined with 25–30 g of carbohydrate intake. We found opposite findings, as we found that mycoprotein, similarly to soy, independently lowered glucose iAUC0-180 min and did not affect insulin response, however the population used was people with T2D, which could explain the different findings. Recruited participants in the current study may have had different degrees of impaired insulin sensitivity/secretion and had an heterogenous intake of drugs affecting different mechanisms (some on lifestyle only, while others on metformin, gliptins, sulfonylureas) which may have a long-lasting effect on insulin sensitising/stimulating effects, despite asking them to refrain from their morning dose on study visit only. These factors described above may have contributed to greater insulin response variability and lack of significant effect by protein. However, the reduced PG response observed with mycoprotein could be due to both the fibre content and hyphal nature of mycoprotein, affecting digestive processes such as alpha-amylase entrapping by the fungal cell walls, thus delaying starch hydrolysis and later glucose absorption as shown in vitro [14]. Other mechanisms such as increased viscosity [34] and delay in gastric emptying [33], are not yet conclusive, and warrant further investigation.

The independent effects observed with guar gum at lowering PG response are in line with previous studies testing guar gum(6 g)-enriched breads in 38 normal weight subjects, where it is observed a 40% reduction compared to high fibre flour-breads [22]. Another study in 11 subjects without diabetes also showed similar effects with 100 g guar gum/kg bread at 30 min in the postprandial glucose response curve [35]. The effects of guar gum at reducing PG response could be because it is a viscous fibre, well-known to decrease PG in healthy adults and long-term glucose control in T2D [21, 23, 36]. Guar gum non-competitively inhibits alpha-amylase [37] and forms a coating on the starch granule, impairing alpha-amylase action due to its increased viscosity [24]. While its effects on gastric emptying have been not proven [38], delayed gastric emptying leads to slowed carbohydrate breakdown, absorption in the gut to bloodstream, and ultimately a reduced PG response.

The significantly increased PG response following a mixed meal in the South Asian vs*.* white European cohort is aligned with previous studies conducted in participants without T2D [39]. A study administering a 5-day dietary challenge mimicking Western diets (high fat, high-calorie diet) showed that healthy South Asians exhibited increased fasting glucose, insulin and insulin resistance compared to age and BMI matched-white European counterparts [7]. Furthermore, another study in metabolically healthy participants showed that the PG response to a malted wheat cereal meal was 62% higher in South Asian participants compared to matched-white Europeans [8]. In our study, we observed an increase in PG response in the South Asian group despite white Europeans having similar age, sex ratio, HbA1c, duration of T2D, and fat free mass ratio. Interestingly, white Europeans had worst metabolic phenotype such as increased body fat, body weight, blood triglycerides, and fasting glucose levels. Although the reason for this differing effect remains unclear, it may be attributed to variations in body fat distribution or the use of medications for T2D —areas that were beyond the scope of this study.

Interestingly, there was a significant interactive effect between guar gum and ethnicity, where the impact of guar gum at lowering postprandial insulin response was only observed in South Asian participants. Decreasing insulin demand from beta-cells may be protective in people with T2D, as hyperinsulinemia overtime is followed by pancreatic beta-cell impairment and exogenous insulin dependence [40]. This study is noteworthy as it utilizes a realistic food product, chapati, which is a flour-based staple in South Asian culture. We demonstrated that the simple addition of 5 g of guar gum, a culturally recognized ingredient, per 100 g of flour, can provide specific benefits for this cohort in managing diabetes. The observed interactive effect was unexpected; we did not anticipate differences in insulin response among ethnicities due to guar gum, which primarily influences digestive processes, such as reducing alpha-amylase activity. The underlying reasons for this interactive effect remain unclear, as this study was not designed to investigate the mechanisms responsible for the varying physiological responses.

Gut hormone response, *ad libitum* and post-visit energy intake were not affected by protein type, guar gum or ethnicity. This finding is in contrast with the literature on the effects on energy intake by mycoprotein using similar amounts in metabolically healthy cohorts [10, 41]. Our observation that the addition of guar gum to the test meals did not result in a significant impact on *ad libitum* energy intake is in line with a recent meta-analysis also showing no effect of guar gum-enriched foods on energy intake, and explained by a loss of satiating properties when guar gum is given in the form of bread compared to liquid [42].

The main strength of this study is its design (acute, controlled, randomised) and the tight control for glycaemic confounders (i.e., standard evening meals, strenuous physical activity avoidance, iso-caloric and -macronutrient matched meals). This allowed us to dissociate, as much as possible, the effect of added ingredients being investigated (protein type or guar gum) on postprandial glycaemic responses. Furthermore, half of the participants were of South Asian ethnicity, allowing for comparison of PG responses between ethnic groups with culturally relevant foods and ingredients (chapati, guar gum, mince protein format) and pave the way for generating further evidence to inform south-Asian specific nutritional advice. Importantly, test meals designed for this study delivered approximately 19 g of dietary fibre in one serving, with a good palatability profile, representing >60% of the daily recommended intake of fibre by World Health Organisation’s guidelines.

We recognise the limitation of using a 180-min time frame to capture postprandial metabolic responses in a population with impaired glucose management, rather than the complete postprandial time frame (360-min). This approach may have not been enough to understand the full digestion and metabolic interplay beyond the time of returning to baseline, thereby potentially missing a later-stage effect in postprandial responses to the test meals. We acknowledge that the medication heterogeneity present in this study may have led to reduced interpretability of results. Implementing a more stringent exclusion criterion, such as including only first-line medication for diabetes (e.g., metformin), would homogenize the cohort, reduce variability, minimise further confounding, and allow for a more accurate attribution of changes in postprandial glucose response to the interventions. We also recognise that the number of subjects we recruited is slightly under what we aimed to (*N* = 12 per ethnicity) for unfounded studies. This was because this clinical trial was impacted by the COVID-19 pandemic, time and budget constraints. However, we strongly believe that the variance and effect size hereby reported can help power future nutritional clinical studies using this understudied cohort.

In conclusion, this study shows that replacing chicken for mycoprotein and including guar gum (5 g within 100 g of chapati bread) have additive effects at decreasing PG response in people with T2D. This study also adds on to the current scarce evidence on postprandial responses to culturally adapted mixed meals in South Asians with T2D, who have a poor metabolic response to foods. Nonetheless, this study has only assessed the acute postprandial response, and data from this study may be used to inform the design and power of forthcoming studies aimed at testing chronic combinatory effect of mycoprotein and guar gum on long-term glycaemic control. Moreover, future studies should use a more homogenous cohort to investigate the mechanisms underpinning these effects, such as the role of gut microbiota fermentation, gastric emptying, or gut hormone response, as these remain unclear.

## Supplementary information

Online supplemental material

## Data Availability

All data generated or analysed during this study are included in this published article [and its supplementary information files].
