# Supplementary material for: Investigating the effects of mycoprotein and guar gum on postprandial glucose in type 2 diabetes: a double-blind randomised controlled trial
Source: Nutr Diabetes. 2025 May 23;15:23. doi: 10.1038/s41387-025-00375-w (PMC12102162; doi:10.1038/s41387-025-00375-w)
Supplement: Supplementary file 1 — Online supplemental material [file 41387_2025_375_MOESM1_ESM.docx]

**SUPPLEMENTAL MATERIAL**

| **Supplemental Table 1.** Nutritional composition of mycoprotein (raw ingredient), Mycoprotein (processed, as sold) and chicken breast per 100g. | | | |
| --- | --- | --- | --- |
|  | **Mycoprotein, raw ingredient (dry weight)^1^** | **Mycoprotein, processed**  **(wet weight)^2^** | **Chicken breast, skinless**  **(wet weight)^3^** |
| **Energy (kcal/100g)** | 340 | 85 | 137 |
| **Protein (g/100g)** | 44 | 11 | 30 |
| **Carbohydrate (g/100g)**  **Of which sugars (g/100g)** | 36  0 | 9  0 | 0 0 |
| **Fat (g/100g)**  **Of which saturated (g/100g)** | 12  3 | 3  0.7 | 1.6  0.5 |
| **Fiber (AOAC) (g/100g)** | 24 | 6 | 0 |
| AOAC, Association of Analytical Chemists; G, gram; kcal, kilocalories.  1. Finnigan T, Needham L, Abbott C. Mycoprotein: a healthy new protein with a low environmental impact. In: Sustainable Protein Sources. Elsevier. 2017; 305-325.  2. Denny, A., B. Aisbitt, J. Lunn. Mycoprotein and health. Nutrition Bulletin. 2008; 33.4(8): 298-310.  3. Sainbury's whole chicken breast. Available at: https://www.sainsburys.co.uk/gol-ui/product/sainsburys-chicken-fillets-1kg. Accessed: May 2025 | | | |

| **Supplemental Table 2.** Subjective appetite feelings questions in a visual analogue scale of 100 mm. |
| --- |
| - Hunger (“How hungry do you feel right now?”) - Fullness (“How full do you feel right now?”) - Thirst (“How thirsty do you feel right now?”) - Desire to eat (“How strong is your desire to eat?”) - Nausea (“How sick do you feel right now?”) - Prospective food intake (“How strong is your appetite for a meal?”) - Appetite for a snack (“How strong is your appetite for a snack?”) - Appetite for savoury (“How strong is your appetite for something savoury?”) - Appetite for sweet (“How strong is your appetite for something sweet?”) |

| **Supplemental Table 3.** Nutritional profile of the non-GG enriched chappati. | |
| --- | --- |
|  | 100g |
| Energy (kcal) | 125.75 |
| Fat (g) | 2.62 |
| Saturates (g) | 0.39 |
| Carbohydrate (g) | 15.92 |
| Starch (g) | 12.86 |
| Sugars (g) | 3.00 |
| Fibre (g) | 3.27 |
| Protein (g) | 8.00 |
| G, gram; GG, guar gum. | |

| **Supplemental Table 4.** Coefficient of variability of assays | | | | |
| --- | --- | --- | --- | --- |
| CV | Glucose | Insulin | PYY | GLP1 |
| Inter-visit variability | 9.5% | 25.5% | 39.5% | 29.4% |
| Inter-assay | 3.6% | 5.0% | 5.0% | 5.0% |
| CV, coefficient of variability; GLP-1, glucagon like peptide-1; PYY, peptide tyrosine tyrosine. | | | | |

| **Supplemental Table 5.** Fasting values for each metabolite. | | | | | | | |
| --- | --- | --- | --- | --- | --- | --- | --- |
|  |  | Chicken | Mycoprotein | Soy | Chicken GG | Mycoprotein GG | Soy GG |
| Glucose  (mmol/L) | Mean | 7.85 | 6.97 | 7.43 | 7.19 | 7.59 | 7.31 |
|  | SD | 1.74 | 1.26 | 1.29 | 1.14 | 1.77 | 1.39 |
| Insulin  (uU/mL) | Mean | 13.58 | 11.15 | 11.43 | 13.41 | 15.42 | 12.24 |
|  | SD | 5.98 | 5.52 | 5.13 | 9.41 | 7.12 | 3.64 |
| PYY  (pmol/L) | Mean | 28.55 | 31.82 | 24.12 | 24.74 | 27.80 | 24.61 |
|  | SD | 17.93 | 36.95 | 25.49 | 24.88 | 25.92 | 22.61 |
| GLP-1  (pmol/L) | Mean | 31.45 | 37.00 | 34.05 | 36.57 | 34.41 | 38.20 |
|  | SD | 15.14 | 18.09 | 17.50 | 22.17 | 21.27 | 23.62 |
| GG, guar gum. GLP-1, glucagon like peptide-1; PYY, peptide tyrosine tyrosine; SD, standard deviation. | | | | | | | |

| **Supplemental Table 6.** Baseline food intake of 21 participants per day (11 white European and 10 south Asian) | | | | | | |
| --- | --- | --- | --- | --- | --- | --- |
|  | **All**  **(N=21)** | | **White European (n=11)** | | **South Asian (n=10)** | |
|  | **Mean** | **SEM** | **Mean** | **SEM** | **Mean** | **SEM** |
| **Energy (kcal)** | 1799 | 71.70 | 1746 | 103.14 | 1687 | 200.26 |
| **CHO (g)** | 208.08 | 11.83 | 194.71 | 18.13 | 203.80 | 24.45 |
| **Total Sugars (g)** | 65.72 | 5.76 | 63.17 | 7.25 | 62.68 | 10.92 |
| **CHO (%)** | 46 | 2 | 45 | 70 | 48 | 49 |
| **Fat (g)** | 77.00 | 5.01 | 75.25 | 6.28 | 71.85 | 10.75 |
| **Fat (%)** | 38 | 2 | 39 | 55 | 38 | 48 |
| **SFA (g)** | 27.44 | 2.08 | 30.53 | 3.27 | 21.62 | 2.78 |
| **CN6-PUFA (g)** | 7.49 | 1.94 | 7.59 | 3.25 | 6.96 | 2.02 |
| **CN3-PUFA (g)** | 1.07 | 0.26 | 1.09 | 0.46 | 0.99 | 0.18 |
| **MUFA (g)** | 25.08 | 1.77 | 25.28 | 2.43 | 22.60 | 3.42 |
| **PUFA (g)** | 12.89 | 1.32 | 11.21 | 1.43 | 13.60 | 2.52 |
| **Trans Fats (g)** | 0.97 | 0.11 | 1.11 | 0.15 | 0.74 | 0.16 |
| **Protein (g)** | 75.05 | 2.63 | 75.30 | 3.93 | 67.68 | 7.87 |
| **Protein (%)** | 17 | 1 | 17 | 15 | 16 | 16 |
| **Fiber (g)** | 20.41 | 1.15 | 18.94 | 1.51 | 20.14 | 2.60 |
| **Starch (g)** | 138.32 | 8.22 | 128.08 | 12.40 | 137.00 | 16.60 |
| **Alcohol (g)** | 3.49 | 1.95 | 4.83 | 3.38 | 1.99 | 1.60 |
| CHO, carbohydrate; MUFA, monounsaturated fatty acids; PUFA, polyunsaturated fatty acids; SEM, standard error of the mean; SFA, saturated fatty acids. T-test performed between white European and south Asian. P-value ≤0.05 is considered significant. | | | | | | |


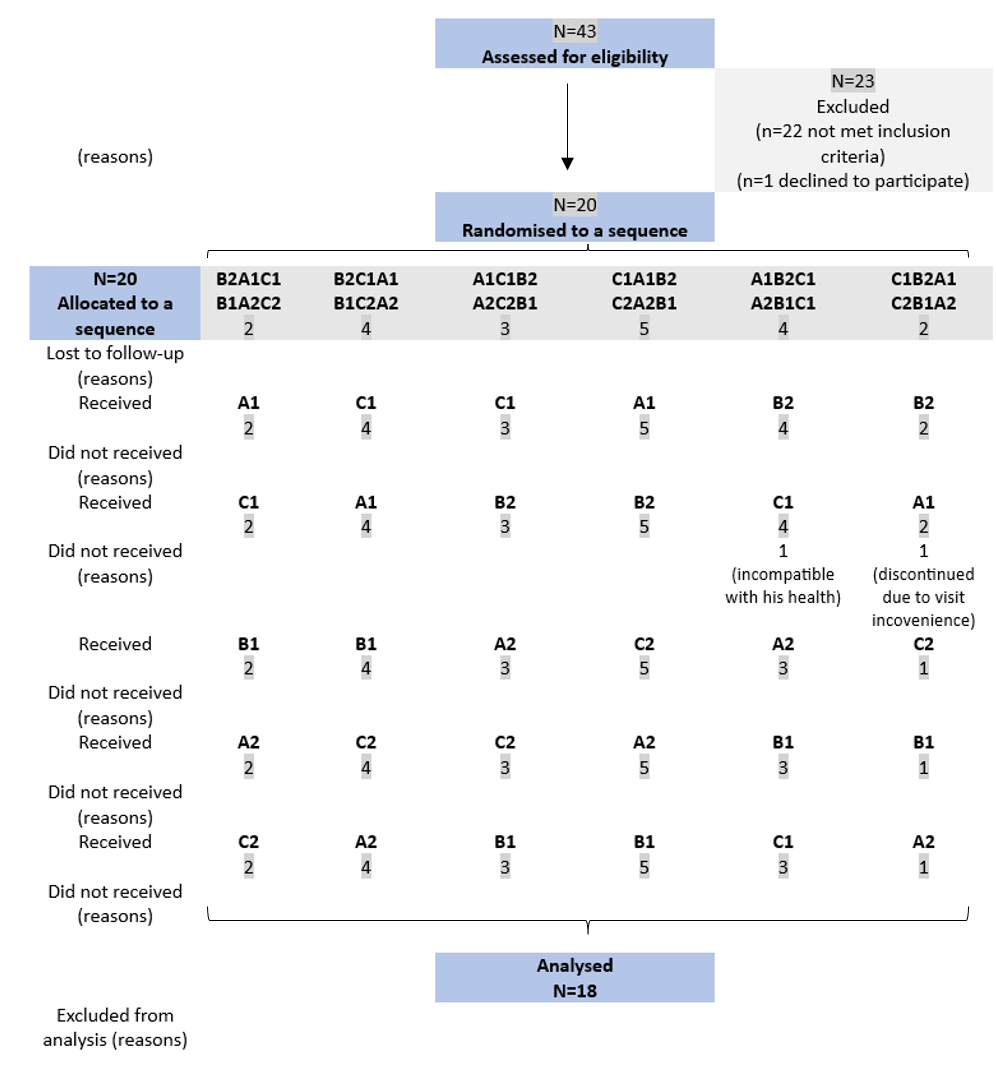


**Supplemental Figure 1** Consolidated Standards of Reporting Trials (CONSORT) diagram of the flow of subjects through the cross-over study (Dawn et al., 2019). N, sample size. A, chicken, B, mycoprotein, C, soy, 1, plain chapati, 2, guar gum-enriched chapati.

Reference: Dwan, Kerry, et al. "CONSORT 2010 statement: extension to randomised crossover trials." *bmj* 366 (2019).

| **A 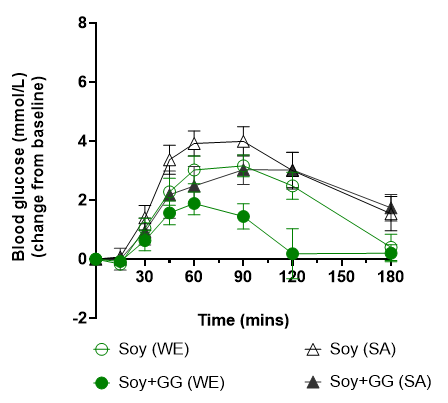** | |
| --- | --- |
| **B**  **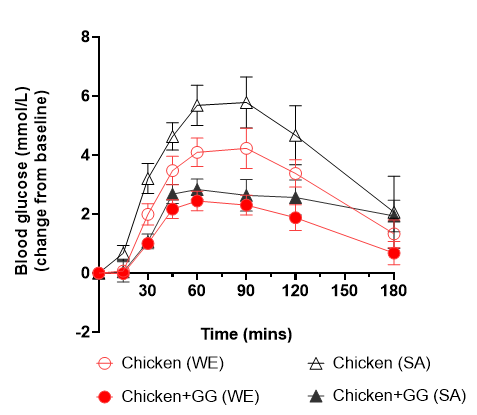** | **C**  **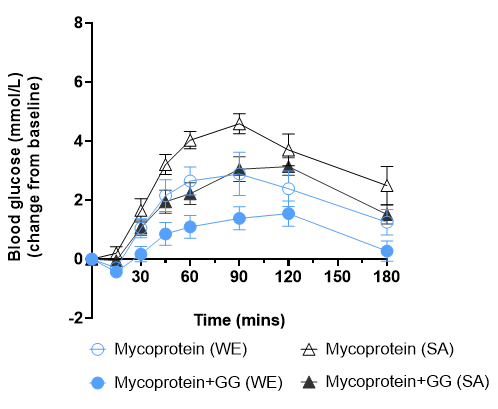** |

**Supplemental Figure** **2. Plasma glucose change from baseline concentration over 180 min following the consumption of soy (A), chicken (B) and mycoprotein (C) with GG- or not enriched chapatis by ethnicity (total n=18; 10 white Europeans and 8 south Asians)**. Test food was given at timepoint 0 min. Data represents mean±SEM. The colour scheme represents the type of proteins, these being soy (green), chicken (red), and mycoprotein (blue). The filling of the figures represents the type of chapati, these being plain chapatis (empty circles or triangles) and GG-enriched chapatis (filled circles or triangles). Triangles are south Asian and circles are white Europeans. GG, guar gum; SA, south Asians; SEM, standard error of mean; n, sample size; WE, white Europeans.

| **A**  **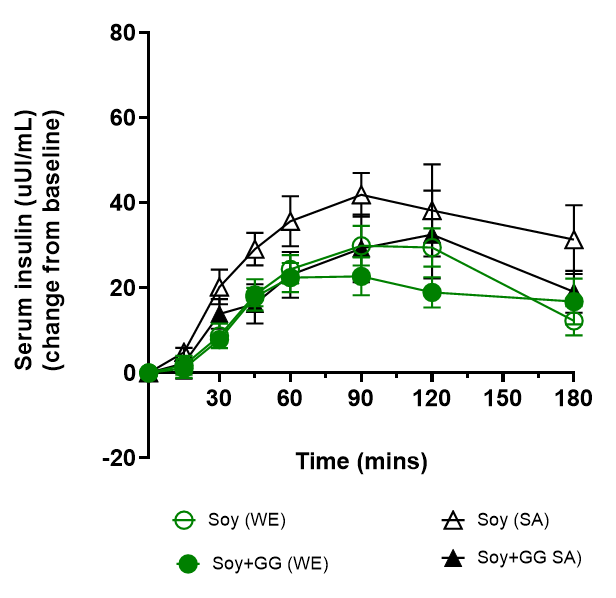** | **B**  **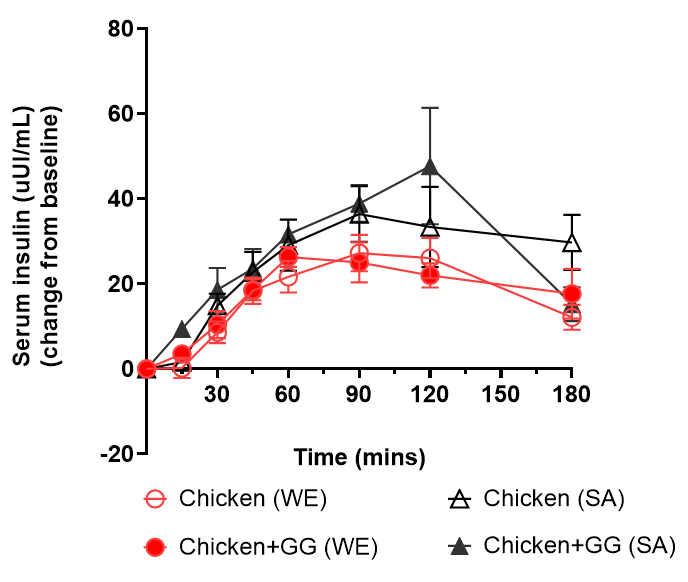** |
| --- | --- |
| **C**   | |

**Supplemental Figure 3. Serum insulin change from baseline concentration over 180 min following the consumption of soy (A), chicken (B) and mycoprotein (C) with GG- or not enriched chapatis by ethnicity (total n=18; 10 white Europeans and 8 south Asians)**. Test food was given at timepoint 0 min. Data represents mean±SEM. The colour scheme represents the type of proteins, these being soy (green), chicken (red), and mycoprotein (blue). The filling of the figures represents the type of chapati, these being plain chapatis (empty circles or triangles) and GG-enriched chapatis (filled circles or triangles). Triangles are south Asian and circles are white Europeans. GG, guar gum; SA, south Asians; SEM, standard error of mean; n, sample size; WE, white Europeans.

**
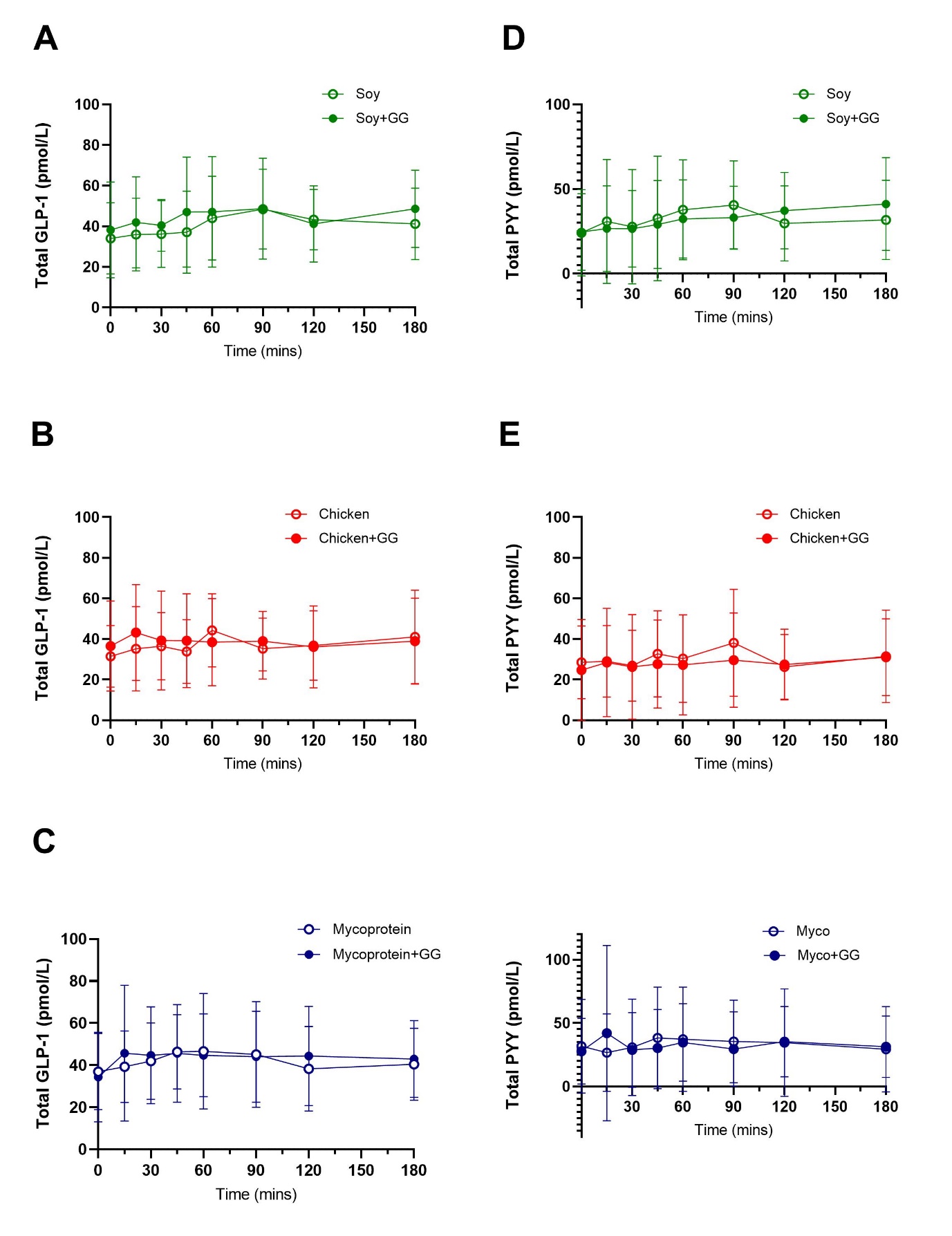
Supplemental Figure 4. Plasma total GLP-1 and PYY concentration over 180 min following the consumption of soy, chicken and MP with GG- or not enriched chapatis for all participants (n=21)**. Total GLP-1 (**A,B,C**) in which **A** Soy with and without GG-enriched chapati. **B** Chicken with and without GG-enriched chapati. **C** Mycoprotein with and without GG-enriched chapati, and Total PYY (**D,E,F**) in which **D** Soy with and without GG-enriched chapati. **E** Chicken with and without GG-enriched chapati. **F** Mycoprotein with and without GG-enriched chapati**.** Test food was given at timepoint 0 min. Data represents mean±SD. The colour scheme represents the type of proteins, these being soy (green), chicken (red), and MP (blue). The filling of the circles represent the type of chapati, these being plain chapatis (empty circles) and GG-enriched chapatis (filled circles). GG, guar gum; GLP-1, glucagon like peptide-1; SD, standard deviation; MP, mycoprotein; n, sample size; PYY, peptide tyrosine tyrosine.

| **A 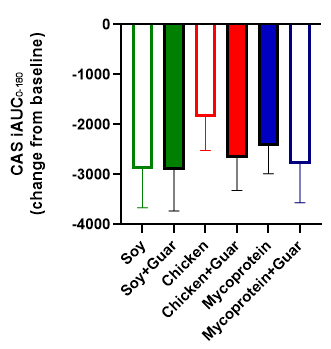** | **B 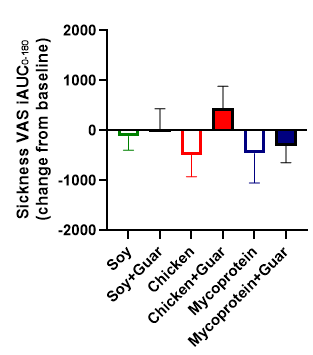** |
| --- | --- |

**Supplemental Figure 5. Composite appetite score (CAS) and sickness VAS change from baseline incremental AUC0-180 following consumption of soy, chicken, and mycoprotein with or without guar gum for all participants (both south Asians and white Europeans pooled) (n=18). A** Incremental area under the curve (AUC) of the change from baseline 0-180 min of the composite appetite score (CAS) following the ingestion of soy, chicken, and mycoprotein with or without GG. Data represents mean±SD. B Incremental area under the curve (AUC) of the change from baseline 0-180 min of the sickness VAS following the ingestion of soy, chicken, and mycoprotein with or without GG. Data represents mean±SD. Test food was given at timepoint 0 min. The colour scheme represents the type of proteins, these being soy (green), chicken (red), and mycoprotein (blue). In the timeline graph, the geometry represents the type of proteins, these being soy (triangle), chicken (circle), and mycoprotein (square). The filling of the geometric datapoints represent the type of chapati, these being without guar gum (empty) and with guar gum (filled). A linear mixed models for independent effects of protein, guar gum and ethnicity and for the interaction between them was performed and showed no significances. AUC, area under the curve; CAS, composite appetite score; GG, guar gum; n, sample size; SD, standard deviation; VAS, visual analogue scale.

**
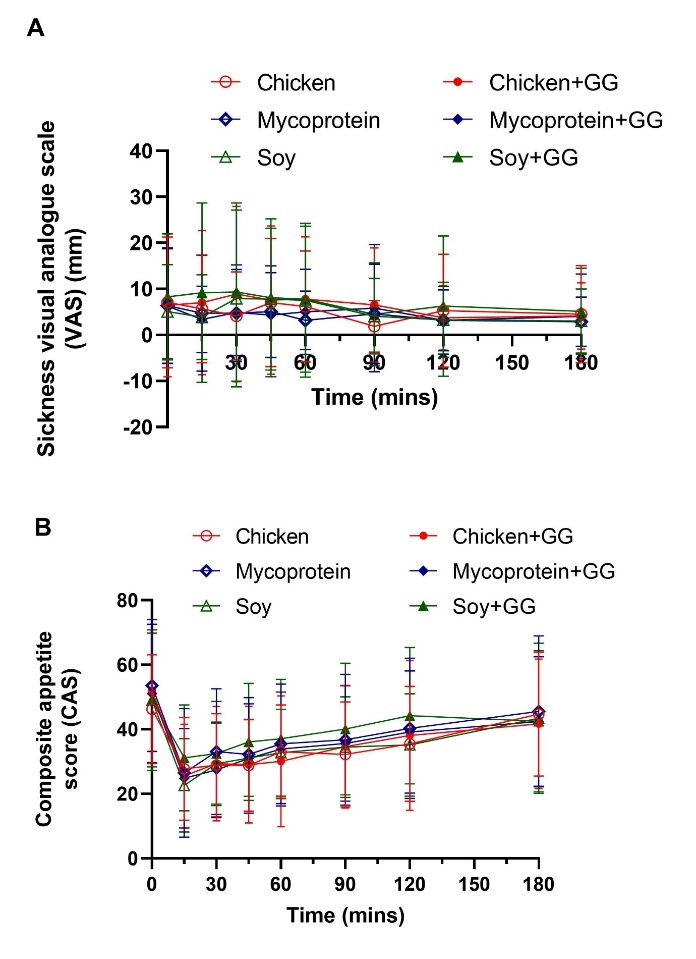
**

**Supplemental Figure 6. Sickness visual analogue scale (VAS) (A) and composite appetite score (CAS) (B) ratings (mm) over 180 min following the consumption of soy, chicken and MP with GG- or not enriched chapatis for all participants (n=21)**. Data represents mean±SD. The colour scheme represents the type of proteins, these being soy (green), chicken (red), and MP (blue). CAS, composite appetite score; GG, guar gum; SD, standard deviation; mm, millimetres; MP, mycoprotein; n, sample size; VAS, visual analogue scale.
